# Supplementary material for: Short-End Injection Capillary Electrophoresis and Multivariate Analysis for Simultaneous Determination of Heavy Metals in Passiflora incarnata Tea
Source: Int J Environ Res Public Health. 2022 Nov 30;19(23):15994. doi: 10.3390/ijerph192315994 (PMC9735907; doi:10.3390/ijerph192315994)
Supplement: Supplementary file 1 [file ijerph-19-15994-s001.zip › ijerph-2061126-supplementary.pdf]

Supplementary Materials

Figures

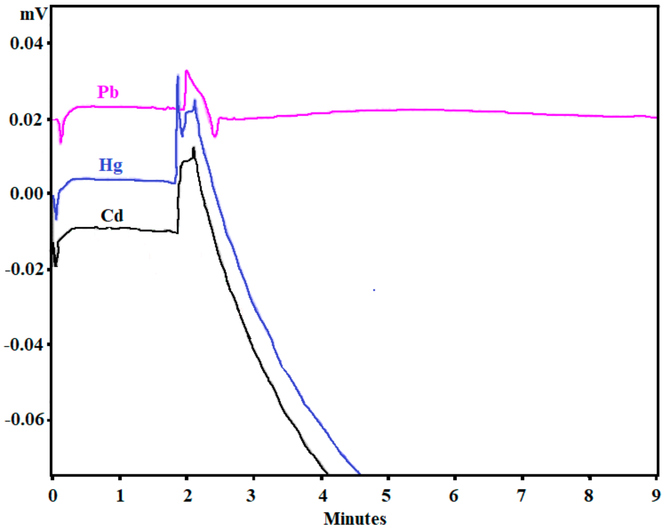

Figure S1. Electropherograms for Cd^{2+}, Pb^{2+} and Hg^{2+} separation using lactic acid as complexant agent. Conditions: indirect mode, 38.8 cm effective length, 50 cm full length capillary, voltage 10 kV, BGE 15 mmol $^{L-1}$ lactic acid, 5 mmol L^{-1} imidazole, 20% methanol, temperature at 15 °C, sample injection at 0.5 psi by 5 s and detection at 214 nm.

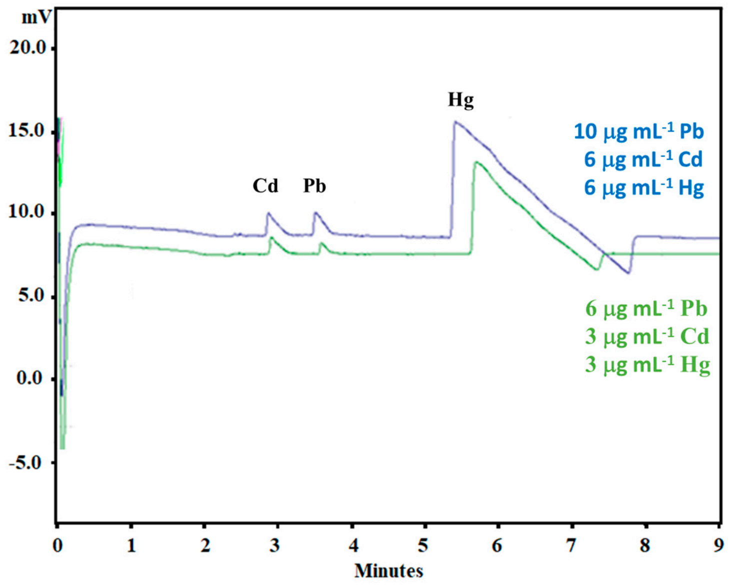

Figure S2. Electropherograms for Cd^{2+}, Pb^{2+} and Hg^{2+} separation using long-term injection. Conditions: indirect mode, 38.8 cm effective length, 50 cm full-length capillary, BGE with 5 mmol L^{-1} imidazole, 6 mmol L^{-1} HIBA, 50.0 mmol L^{-1} 18-crown-6, and 20% methanol, temperature at 15 ºC, sample injection at 0.5 psi by 5 s and detection at 214 nm.
